# Niche suitability and spatial distribution patterns of anurans in a unique Ecoregion mosaic of Northern Pakistan

**DOI:** 10.1371/journal.pone.0285867

**Published:** 2023-06-15

**Authors:** Muhammad Rais, Muhammad Ali Nawaz, Russell J. Gray, Waqas Qadir, Syeda Maria Ali, Muhammad Saeed, Ayesha Akram, Waseem Ahmed, Anum Sajjad, Lionel Leston

**Affiliations:** 1 Department of Zoology, Herpetology Lab, Wildlife and Fisheries, Pir Mehr Ali Shah Arid Agriculture University Rawalpindi, Rawalpindi, Pakistan; 2 Department of Biological and Environmental Sciences, Environmental Science Program, College of Arts and Sciences, Doha, Qatar; 3 Science Advisor, Save Vietnam’s Wildlife, Ninh Bình, Vietnam; 4 Assistant Education Officer, Rawalpindi, Pakistan; 5 Department of Environmental Sciences, International Islamic University Islamabad, Islamabad, Pakistan; 6 Research & Planning Wildlife, Islamabad Wildlife Management Board (IWMB), Ministry of Climate Change, Islamabad, Islamabad; 7 Department of Zoology, Wildlife and Fisheries, Pir Mehr Ali Shah Arid Agriculture University Rawalpindi, Rawalpindi, Pakistan; 8 Occupational Health Safety and Environment, North West General Hospital and Research Centre, Hayatabad, Peshawar; 9 Department of Biological Sciences, University of Alberta, Edmonton, Alberta, Canada; Bowling Green State University, UNITED STATES

## Abstract

The lack of information regarding biodiversity status hampers designing and implementing conservation strategies and achieving future targets. Northern Pakistan consists of a unique ecoregion mosaic which supports a myriad of environmental niches for anuran diversity in comparison to the deserts and xeric shrublands throughout the rest of the country. In order to study the niche suitability, species overlap and distribution patterns in Pakistan, we collected observational data for nine anuran species across several distinct ecoregions by surveying 87 randomly selected locations from 2016 to 2018 in Rawalpindi District and Islamabad Capital Territory. Our model showed that the precipitation of the warmest and coldest quarter, distance to rivers and vegetation were the greatest drivers of anuran distribution, expectedly indicating that the presence of humid forests and proximity to waterways greatly influences the habitable range of anurans in Pakistan. Sympatric overlap between species occurred at significantly higher density in tropical and subtropical coniferous forests than in other ecoregion types. We found species such as *Minervarya* spp., *Hoplobatrachus tigerinus* and *Euphlyctis* spp. preferred the lowlands in proximal, central and southern parts of the study area proximal to urban settlements, with little vegetation and higher average temperatures. *Duttaphrynus bengalensis* and *D*. *stomaticus* had scattered distributions throughout the study area with no clear preference for elevation. *Sphaerotheca pashchima* was patchily distributed in the midwestern extent of the study area as well as the foothills to the north. *Microhyla nilphamariensis* was widely distributed throughout the study area with a preference for both lowlands and montane terrain. Endemic frogs (*Nanorana vicina* and *Allopaa hazarensis*) were observed only in locations with higher elevations, higher density of streams and lower average temperatures as compared to the other seven species sampled. It is recommended to provide legal protection to amphibians of Pakistan, especially endemic species, through revision in the existing wildlife laws. We suggest studying the effectiveness of existing amphibian tunnels and corridors or designing new ones tailored to the needs of our species to prevent their local extinction due to ongoing or proposed urban development which might affect their dispersal and colonization.

## Introduction

The Wallacean shortfall, which suggests our understanding of geographical distribution patterns of the species at large scales is generally poor, can be minimized by sampling ecosystem gradients at smaller scales and expanding our knowledge outward [1]. Despite some progress in achieving global strategic goals of the United Nations and the Aichi Targets, biodiversity has declined, particularly in developing countries [2]. The lack of information regarding biodiversity status precludes recognition of impacts of anthropogenic activities on biodiversity and implementation of conservation strategies and targets [3]. Therefore, to gain a large-scale understanding of any taxonomic group and to determine threats and conservation potential within their spatial distribution in accordance with environmental variability, there must first be fine-scale (local/regional) baseline data.

Amphibian occurrence and abundance are greatly influenced by localized variation in geomorphic, geologic, and environmental characteristics [4]. Species Distribution Modelling (SDM) approaches based on Geographical Information System (GIS) have been widely used to predict distribution of species of amphibians and other vertebrate groups such as rodents and passerine birds [5–11] for conservation purposes. However, with presence-only records often being the only available data, and potential for imperfect detection within a study location, one must account for distribution and absence given contributing environmental covariates [12,13].

Amphibians in Pakistan have long been ignored in research, conservation, management, and policy and legislation. Pakistan’s National Climate Change Policy (2012), National Biodiversity Strategy and Action Plan (2015) and Pakistan Wetland Action Plan (2000) proposed guidelines for the conservation of natural resources, including fauna and flora, and mitigation of threats [14,15]. Currently, 21 species of amphibian (all anurans) have been documented in Pakistan [16] of which nine are believed to be endemic [17]. However, no progress on integrating anurans in wildlife conservation, and policy development or legislation has been made. There is no national assessment of conservation status of anurans of Pakistan which cautions the use of global conservation status. Only a few published studies report the richness and abundance of various Pakistani anuran species [18–24]. For example, the common Skittering Frog (*Euphlyctis cyanophlyctis*) [18] was reported as abundant in the rice fields of Gujranwala, Punjab Province [19], and nine anuran species were recorded from Margalla Hills National Park, Islamabad [20]. Six anuran species were reported from Rawalpindi and Islamabad areas [21,22] including high abundance of Indus Valley Bull Frog (*Hoplobatrachus tigerinus*) [23] and Skittering Frog from Rawal Lake, Islamabad [24].

In the current study, we aimed to estimate niche suitability, niche overlap and model distribution of anuran species within the Rawalpindi District and Islamabad Capital Territory, which encompasses a unique mosaic of deserts and xeric shrublands, montane grasslands, temperate broadleaf and mixed forests, temperate conifer forests, and tropical/subtropical coniferous forests [25]. Our findings were expected to generate new information on factors affecting niche suitability, spatial distribution size, and important ecoregions of the studied species and for anuran diversity to inform future conservation plans in Pakistan.

## Materials and methods

### Study area

The study was carried out in seven administrative units (Gujar Khan, Kahuta, Kallar Sayedan, Kotli Sattian, Murree, Rawalpindi, and Taxila) of the Rawalpindi District and Islamabad Capital Territory, Pakistan. The Rawalpindi District (33.4620° N, 73.3709° E) is located in the north-west of Punjab Province and covers an area of 5312 km^2^. Islamabad Capital Territory (ICT) (33.7205° N, 73.0405° E) is in the north-east of the country and covers an area of 906.50 km^2^. The elevation ranges from 457–2286 m and 457–610 m in Rawalpindi District and Islamabad Capital Territory, respectively. The climate of the study area is humid subtropical (Koppen climate classification). The summers produce more rain than the winter due to the monsoon season (July-August). The average rainfall in Islamabad Capital Territory is about 940 mm, in areas of Rawalpindi, Gujar Khan and Taxila ranges from 970–990 mm and in Murree, Kotli Sattian, and Kahuta is 1249 mm [26–28].

**Fig 1:**
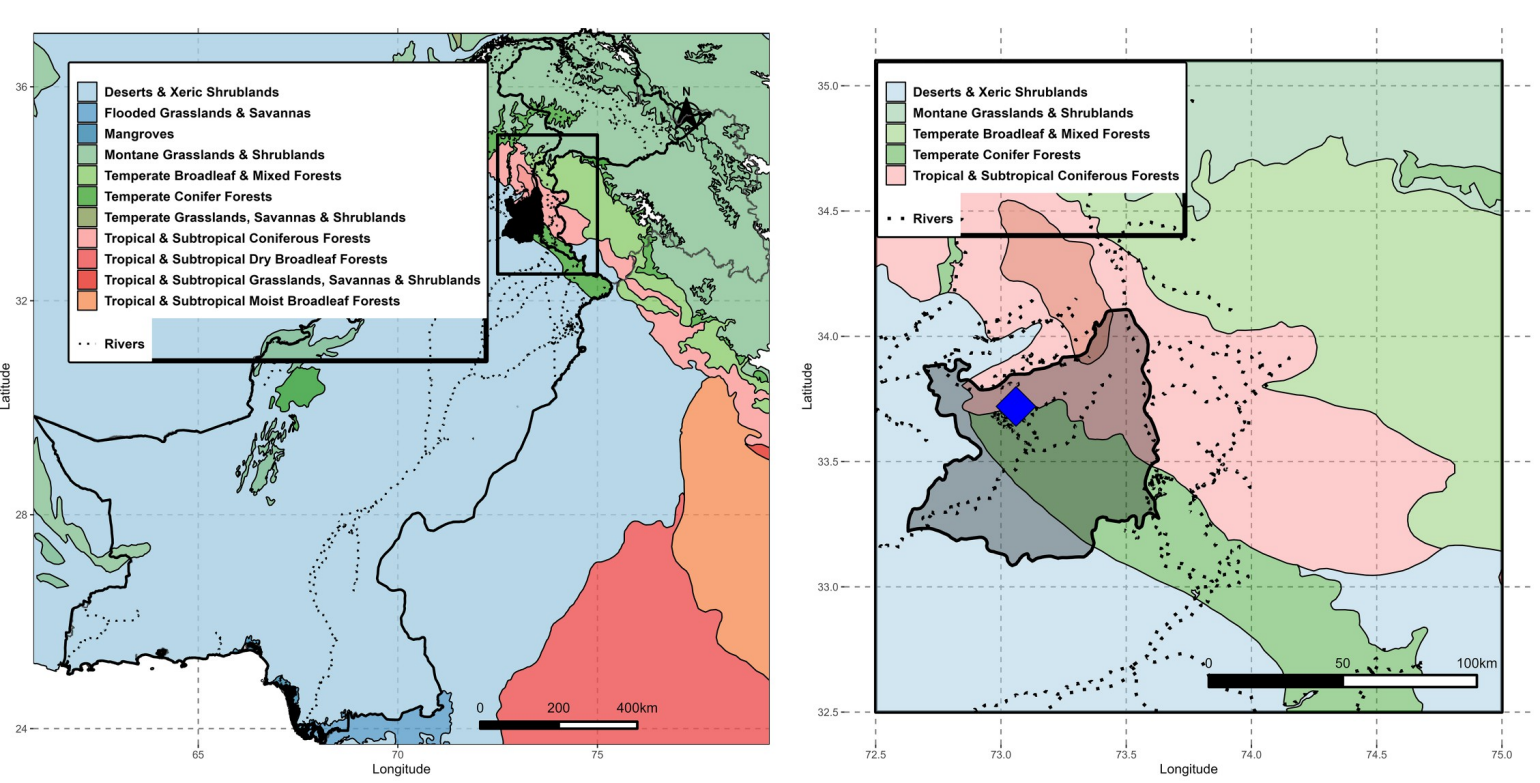
Map showing eleven major ecoregions in Pakistan (left) and ecoregions present in the extent of the present study area (right). The study area is shown with black boundary and grey shaded area while dotted lines represent river and watershed distributions throughout the areas. The study area is dominated by tropical and subtropical coniferous forests in the north; broad-leaf and mixed forests, montane grasslands, and montane shrublands in the mid and midwest; and arid shrublands in the east and south. The capital city Islamabad (shown as a blue box) is located in the midwestern portion of the study area. Ecoregion boundaries obtained from RESOLVE Ecoregions and Biomes database (Bioscience, An Ecoregions-Based Approach to Protecting Half the Terrestrial Realm DOI: https://doi.org/10.1093/biosci/bix014), available in ArcGIS Online under a CC by 4.0 license.

The study area is dominated by tropical and subtropical coniferous forests in the north; broad-leaf, mixed forest and montane grasslands and shrublands in the mid and midwest; and arid shrublands in the east and south (Fig 1). The tropical and subtropical coniferous forests are dominated by *Pinus wallichiana* and *Pinus roxburgii* and have relatively fewer human settlements. The proximal, central and southern regions feature urban and semi-urban areas with vegetation species such as *Acacia modesta*, along with *Olea cuspidata*, and *Dodonea viscosa* [29].

Of the 9 anuran species that are endemic to Pakistan [17], and therefore are of increased conservation interest, our study area encompasses most of the range of two of these endemics within Pakistan. Hazara Torrent Frog (*Allopaa hazarensis*) [30] is endemic to the springs and streams of Northern Pakistan [30]. Murree Hills Frog (*Nanorana vicina*) [31] is endemic to Pakistan and India [32].

### Data collection

We surveyed 87 randomly selected sites in the seven administrative units of Rawalpindi District and Islamabad Capital Territory and gathered anuran presence data from 2016 to 2018 (March–September) on a monthly basis; we visited each site at least twice from during the study period. The duration and number of observers varied during the surveys; however, on average each visit consisted of two days of non-standard field observations from early morning till late night carried out on a weekly basis, with 4–6 observers. Each visit had at least one observer familiar with the identification of anurans of the area. Anuran species richness was recorded using time-constrained visual encounter survey (VES) technique [33]. Observers actively and thoroughly searched the sampling locations for a predefined time (~60–120 min.) in order to record the species. Adult specimens and tadpoles were collected with dip nets or simply picked up by hand, were examined, and identified following descriptive identification by Khan [29] and released back.

### Data analysis and species distribution modelling

All analyses, models, maps and plots were generated using R statistical software version 3.6.3 [34]. A preliminary check on observed environmental values for each species was conducted by using the extract function in the ‘raster’ package [35]. Environmental ranges of species were checked before running models in order to identify potential flaws in the output (e.g. high spatial distribution probability in montane ranges when the species observations have initial low-elevation values). Bioclimatic variables (BIO 1–19) were retrieved from the WorldClim repository [36] via the ‘raster’ package at 0.5 arcmin resolution, and were tested for collinearity using Variation Inflated Factors (VIF). The following bioclimatic variables were retained: BIO7: temperature (C°) annual range; BIO8: mean temperature (C°) of wettest quarter (3 consecutive months); BIO9: mean temperature (C°) of driest quarter; BIO15: precipitation seasonality; BIO18: precipitation (mm) of the average warmest quarter; and BIO19: precipitation (mm) of the average coldest quarter. To map vegetation, we used MODIS (https://developers.google.com/earthengine/datasets/catalog/MODIS_006_MOD13Q1#bands; https://code.earthengine.google.com/e3a10b1ec6086c3ee7c598cfaca7dd98; resolution: 250m; scale 0.0001). MODIS has a spatial resolution of 250 m and provides a Vegetation Index (VI) value at a per pixel basis. We used two primary vegetation layers: Normalized Difference Vegetation Index (NDVI), which is the continuity index to the existing National Oceanic and Atmospheric Administration-Advanced Very High-Resolution Radiometer (NOAA-AVHRR) derived NDVI; and Enhanced Vegetation Index (EVI), which minimizes canopy background variations and maintains sensitivity over dense vegetation conditions. In Google Earth Engine (GEE), both MODIS layers The MODIS NDVI and EVI products were computed from atmospherically corrected bi-directional surface reflectance that had been masked for water, clouds, heavy aerosols, and cloud shadows. A 30 m Digital Elevation Model (DEM) was retrieved from the USGS earth explorer database (https://earthexplorer.usgs.gov/). The DEM was used to generate slope, aspect, and terrain roughness via the *terrain* function in the ‘raster’ package [37]. Distance to rivers was calculated using a local shape file of all waterways in Pakistan and the *fasterVectToRastDistance* function of the ‘fasterRaster’ package [38]. River distance values (in meters) were log normalized to prevent outlier values from overwhelming the model contribution estimates. All environmental variables were resampled to 0.5 arcmin resolutions thereafter using the bilinear method, and masked to a shapefile of Pakistan regional boundaries. The explanatory variables were then stacked and tested for multicollinearity using the *vifstep* and *vifcor* functions of the ‘usdm’ package [39] with a threshold VIF of 10 and a threshold correlation coefficient with an absolute value of 0.7; whereby we excluded predictors that were strongly correlated with ⸺ but considered to be less important for anurans ⸺ than other predictors.

The Maximum Entropy or “Maxent” modelling method has been shown to perform as well as, or better than ensemble models when modelling species distributions, with additional benefits of lower computational power requirements and increased simplicity of use [40]. Species Distribution Models for this study were created following guidelines on Maxent parameterization [41,42]. Species occurrence data was thinned to one point per raster cell to omit spatial biases. Maxent models were created using the Maxent software wrapper through the ‘dismo’ package [37]. A kernel density bias mask was created by querying all available anuran occurrences from the Global Biodiversity Information Facility (GBIF) database for Pakistan (S1 File) as a measure of restrictive effort using the ‘MASS’ package [43] using the reference bandwidth smoothing factor. We used the *occ_search* function in the ‘rgbif’ package [44] in R to access the GBIF database, using the taxon “Anura”, the country Pakistan (“PK”), and “coordinates = TRUE” as filters to create a text file containing 345 dataset keys and number of observations per dataset) (S2 File). We generated 10,000 geographically randomized background points within the bias mask estimate. Models were tuned using the *ENMeval* function in the ‘ENMeval’ package [45] to identify feature selection variables and regularization multiplier (beta-multiplier) values selected from the lowest delta Akaike Information Criterion (AIC_c_) using five random *k*-folds. Maxent datasets were partitioned into training (3/4) and testing (1/4) data using a 4-way partitioned *k*-fold. Features selected in the final models were linear and quadratic (lq) with a beta-multiplier set to between 1–2 for sensitivity testing, and 1 for the final model. We selected these features for the final Maxent models for each species based on the statistics we used to validate our model: area-under-the-curve (AUC) values within receiver-operating characteristic (ROC) curves and the true skill statistic (TSS [= TP + TN—1, where TP = proportion of true positive predictions and TN = proportion of true negative predictions]). Models were replicated with replacement using the bootstrap method.

Final models were visualized using the raw model output using the ‘ggplot2’ package [46] and each model prediction was condensed using the highest true skill statistics (TSS) threshold value as a filter (hereafter called “threshold model”). The true skill statistics is defined based on the components of the standard confusion matrix representing matches and mismatches between observations and predictions [47]. Model outputs were defined as *niche suitability* on a 0–1 probability scale (1 = highest niche suitability; 0 = lowest niche suitability), and threshold models were considered to be the niche distribution of a given species in terms of overlap with others. Each threshold model was converted to points and extracted from the explanatory variables to derive summary statistics of environmental values for each species within their niche distribution. All species threshold predictions were then combined to create niche overlap raster to determine which eco-regions were most suitable for anuran species in Pakistan. High-density niche overlap areas were identified by filtering 50% (4.5 species per raster cell) and 100% (9 species per raster cell). We then converted the two final niche spaces to a spatial polygon to identify percentage overlap between each ecoregion and high-density niche distributions.

## Results

### Niche suitability and overlap

**Fig 2:**
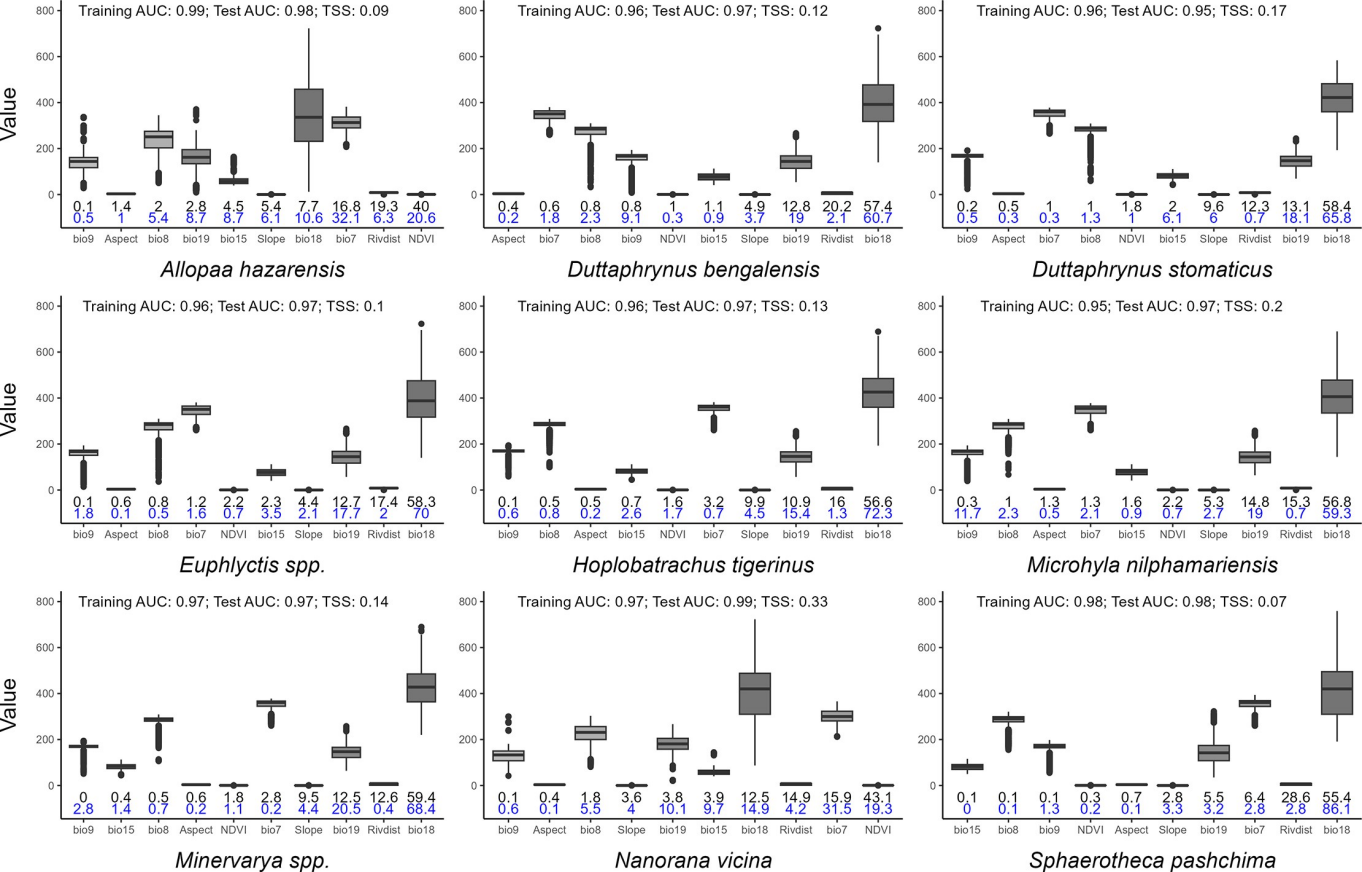
Box plots showing the median, interquartile range, minimum and maximum values of each modelled environmental variable at locations where the nine sampled anuran species in the study area are predicted to be present (i. e., the predicted habitat suitability exceeds the TSS threshold for each species). Numbers above each box plot indicate measures of average model run performance (AUC = area-under-the-curve; TSS = true skill statistic). Numbers below each box plot indicate the percent contribution (top row) and permutation importance (bottom row) of each variable in predicting habitat suitability for each species, averaged over ten model runs per species. BIO7: Temperature (C°) annual range; BIO8: Mean temperature (C°) of wettest quarter; BIO9: Mean temperature (C°) of driest quarter; BIO15: Precipitation seasonality (%); BIO18: Precipitation (mm) of the warmest quarter, BIO19 Precipitation (mm) of the coldest quarter; NDVI: Normalized Difference Vegetation Index; RivDist: (log) Distance to Rivers (m).

Final predictions for niche suitability of all nine anuran species using Maxent models indicated good fit in terms of AUC (Fig 2). Environmental variables varied among species (Fig 2); however, distance to rivers and precipitation in the warmest and coolest quarters were shown to have significant contribution (% contribution >10; higher permutation importance and variable importance in jacknife-testing) (Fig 2) for *Duttaphrynus bengalensis* and *M*. *nilphamariensis;* distance to rivers and precipitation of the warmest quarter for *Duttaphrynus stomaticus;* distance to rivers, precipitation of the warmest quarter, and NDVI for *A*. *hazarensis* and *N*. *vicina*; and precipitation of the warmest quarter and NDVI were most influential for *H*. *tigerinus*, *Euphlyctis* spp., *Minervarya* spp., and *Sphaerotheca pashchima* (S2–S10 Files). Habitat suitability for most species was higher in locations with greater precipitation in the warmest quarter and less precipitation in the coolest quarter. Habitat suitability for *A*. *hazarensis* and *N*. *vicina* was higher in locations with greater ecosystem productivity (higher NDVI) closer to rivers (S3–S11 Files).

### Spatial distribution

**Fig 3:**
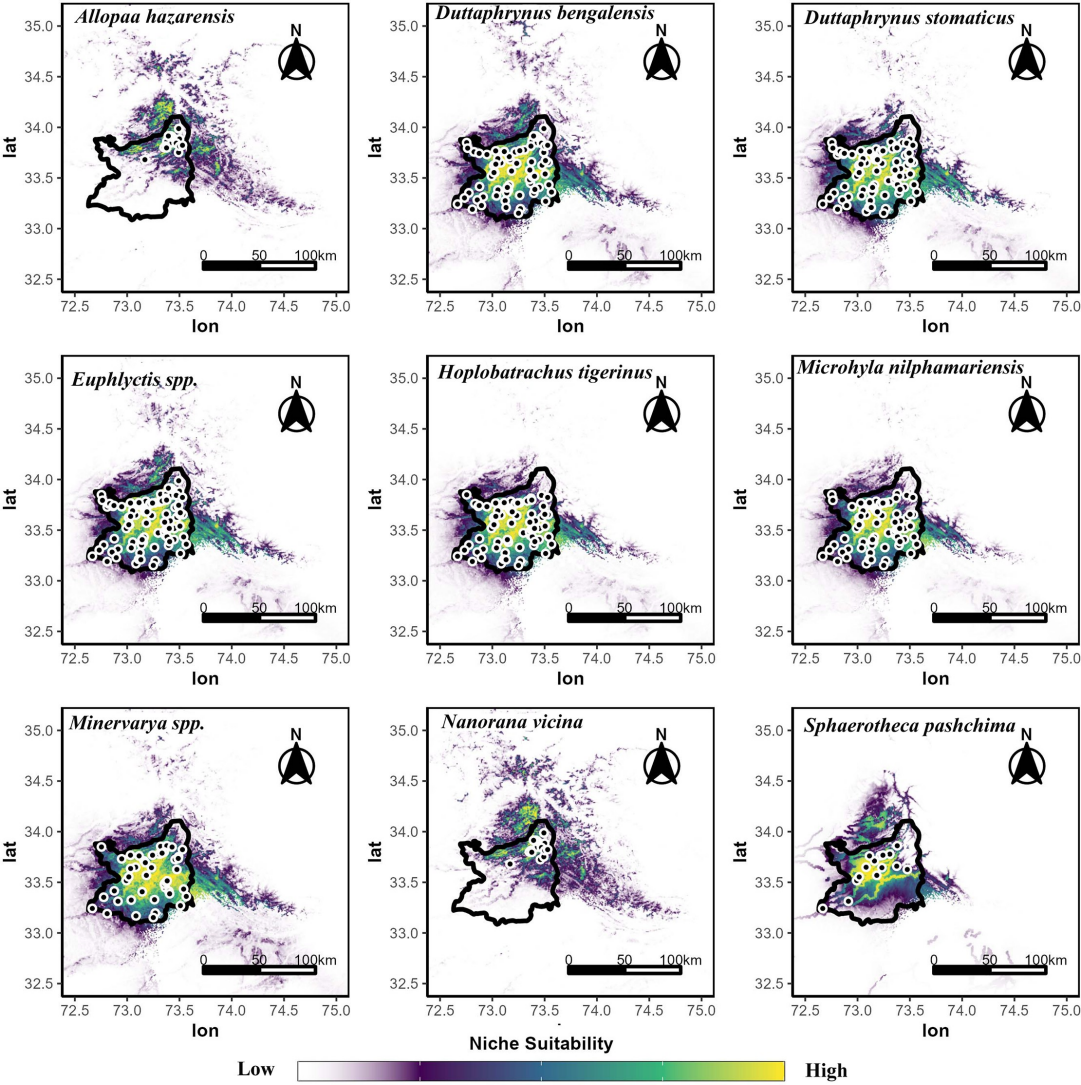
Maxent projections of niche suitability for each of nine anuran species within the study area (area shown as a black polygon while species presence records are represented as small black dots). The predictions were unconstrained which allowed the model to predict outward throughout Pakistan, but predictions mostly stayed in that habitat mosaic in the north.

Nine anuran species showed a divergent pattern of spatial distribution (Fig 3). We found that *Euphlyctis* spp., *Minervarya* spp., and *H*. *tigerinus* showed preference for the lowlands in proximal, central and southern parts of the study with high urban settlements, little vegetation and higher average temperatures. The toads (*D*. *bengalensis* and *D*. *stomaticus*) had scattered distributions throughout the study area with no clear preference for elevation. *S*. *pashchima* showed a patchy distribution in the midwestern extent of the study area as well as the foothills to the north. *Microhyla nilphamariensis* also showed a wide distribution throughout the study area with a preference for both lowlands and montane terrain. Endemic species such as *N*. *vicina* and *A*. *hazarensis* were observed in locations with higher elevations, proximal to streams and lower average temperatures as compared to the other seven species sampled (Fig 3).

**Fig 4:**
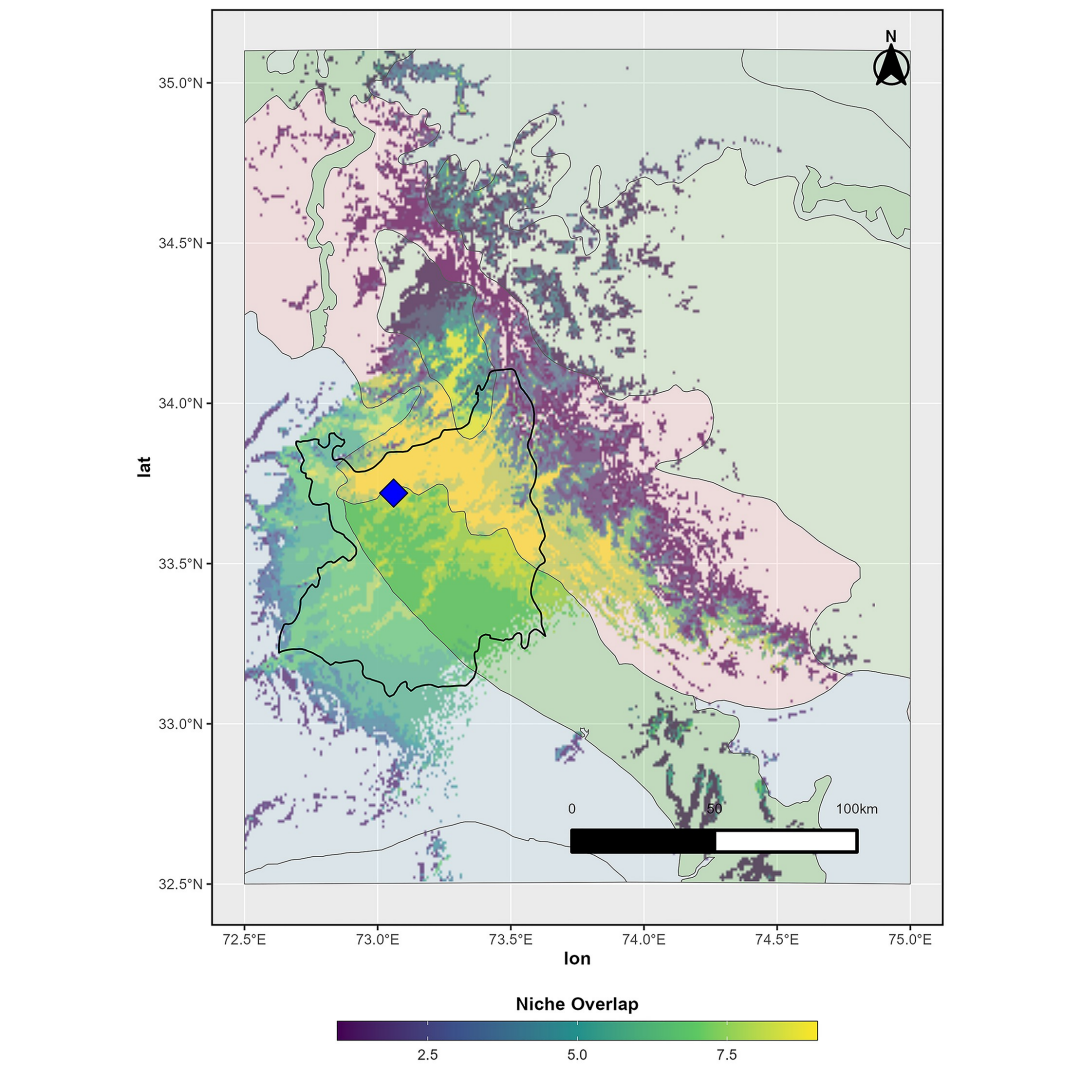
Niche suitability overlap of the nine studied anuran species (combined) within the study area. Lighter areas have more species (richness) and darker areas have less based on suitability predictions above the TSS threshold values for each species. A species was predicted to be present (= 1) if a location’s niche suitability for that species exceeded the TSS threshold for that species; otherwise, the species was predicted to be absent (= 0). Species richness was then summed for each location. Of locations with maximal amphibian species overlap (9 species), 70.2% occurred within tropical/subtropical coniferous forests (pink polygons); 17.0% occurred within temperate coniferous forests (dark green); 9.4% occurred within temperate broadleaf and mixedwood forests (medium green); 3.2% occurred within xeric desert and shrublands (pale blue); and 0% occurred in montane grasslands and shrublands (lightest green). Ecoregion boundaries obtained from RESOLVE Ecoregions and Biomes database (Bioscience, An Ecoregions-Based Approach to Protecting Half the Terrestrial Realm DOI: https://doi.org/10.1093/biosci/bix014), available in ArcGIS Online under a CC by 4.0 license.

Niche overlap at 50% (4.5 species per area) within the studied ecoregions showed 0.2% overlap in montane grasslands and shrublands, 4.8% in temperate broadleaf and mixed forests, 23.6% in temperate coniferous forests and 33.9% in tropical and subtropical coniferous forests. Whereas, the niche overlap (9 species per area) revealed 0% overlap in montane grasslands and shrublands, 3.2% overlap in deserts and xeric shrublands, 9.4% overlap in temperate broadleaf and mixed forests, 17% overlap in temperate coniferous forests, and 70.2% overlap in tropical and subtropical coniferous forests (Fig 4), providing evidence that tropical and subtropical coniferous forests support the highest diversity of anuran species in the region.

## Discussion

Development of analytical habitat distribution models has rapidly increased in ecology due to the invention of new GIS tools and statistical techniques. Such models statistically relate the geographical distribution of species or communities to their present environment. Neither any scientific study on the distribution patterns nor any species occurrence database of anurans of Pakistan exists in the country. In forested mountainous regions outside of but similar to those of Pakistan, amphibians, particularly *Ascaphus truei* and *Dicamptodon tenebrosus*, were more abundant in stream habitats within older coniferous forests [48]. Precipitation and soil temperature influences probability of occurrence for Polish amphibian species [10] while vegetation contributed significantly in the prediction for salamander species of central Portugal [11]. Our results suggest similar trends, with precipitation (of the warmest and coldest quarter), distance to rivers and vegetation being highly deterministic factors of suitability for anurans in Pakistan.

While Maxent models can accurately predict grid-based habitat suitability and presence of species when observation data of those species is limited, predictions from such models could also be used to identify the likeliest locations of species for further monitoring and obtain actual presence/absence or abundance data. Actual presence/absence or abundance data at precise locations enables biologists to use finer-scaled environmental variables that influence habitat suitability and abundance of species. For anurans and other aquatic wildlife, the type of wetland habitat or changes in wetland variables over time at repeatedly monitored sites can increasingly be quantified or classified at fine spatial scales over large regions away from ground-truthed locations [49–54]. Increased availability of wetland data is due to: 1) the accumulation of decades of remotely sensed environmental data by satellites; 2) the development of free, open-source, online platforms (e.g., Google Earth Engine) for processing remotely sensed data and extracting this data to survey locations; and 3) development and sharing of open-source machine learning techniques for predicting and classifying wetlands [49–54]. To improve the chances of detecting anurans or other vocalising species when they are present at sites, monitoring could involve obtaining multiple visits per site by using passive acoustic monitoring with pre-programmed acoustic recorders [55,56]. Acoustic data could then be transcribed to obtain detections per visit and hierarchical models can be used to account for varying detection probability of species among sites when estimating effects of environmental variables on occupancy or abundance [57].

The toads (Family Bufonidae) of the study area: *D*. *bengalensis* and *D*. *stomaticus*, had somewhat scattered distribution throughout the region with no clear preference for elevations; however, *D*. *stomaticus* showed a clear preference to the northwestern extent of the study area, mostly concentrating within and proximal to the lowlands. *D*. *bengalensis* and *D*. *stomaticus* have been recorded as widespread species found up to 1800 m [58] and 4500 m [59] elevations, respectively. *D*. *bengalensis* is adapted to various types of habitats, even degraded ones, and around human habitations [60–62]. The geographic range of *D*. *bengalensis* has now been extended due to its introduction and *D*. *bengalensis* has attained a status of invasive species in various parts of the world [58]. The two toads are found in the plains, lowlands, sub mountain areas as well as hilly areas in Pakistan which experience monsoon season (July-August) during which the species breed [29]. We recorded *D*. *bengalensis* at elevations higher than previously reported. One reason for their widespread distribution in our study area is their adaptation to a wide range of habitats. *M*. *nilphamariensis*, a diminutive frog species (Family Microhylidae), showed a widespread distribution throughout the study area with a preference for both lowlands and montane terrain. The species has been recorded from areas up to 2000 m elevation [63] as well as from lowlands, sub mountain areas and foothills in Pakistan [29].

The dicroglossid frogs of the study area showed varied distribution patterns. *S*. *pashchima*, a burrowing frog species, showed a patchy distribution in the midwestern extent of the study area as well as the foothills to the north. *S*. *pashchima* has been recorded as a widespread species from lowlands and forested areas up to 1500 m [64]. This species remains under soft soil for most parts of the year and emerges during summers to breed during the monsoon [29] and avoids high altitude areas possibly due to their burrowing habit, since hard substrate makes it difficult for them to dig. Further, mountains in the north are under less influence of monsoon, which likely provides less suitable breeding conditions. Other dicroglossid frogs such as *Euphlyctis* spp. showed scattered distribution probability through the lowlands. *Minervarya* spp. was shown to prefer proximal, central, southern, and western lowland areas. *H*. *tigerinus* also showed variability in preference between lowland and elevated areas, with a concentrated distribution toward the middle to northern extents of the study area. *H*. *tigerinus* has previously been recorded from areas up to 2000 m [65]. These areas are amongst most built up parts of the region in addition to encompassing other human modified habitats such as croplands [44,66]. *Euphlyctis* spp. has been recorded from areas up to 2500 m [67] while *Fejervarya* spp. from areas up to 2000 m (Dijk 2004) [58], but these dicroglossid frogs are also widespread in lowlands and forested areas. *Euphlyctis cyanophlyctis* and *Zakerana syhadrensis* occur along stream banks and water pools between forest edges, agricultural areas, and residential gardens [62]. Our findings are consistent with the available information. However, we have thus forth provided empirical data on the response of these species to the studied environmental factors for the first time.

The endemic frogs, *A*. *hazarensis* and *N*. *vicina*, showed restricted occurrence within the northern and north-eastern mountain ranges in areas of high elevation compared to the proximal lowlands to the southeast. Most of these areas feature subtropical pine forest (900–1500 m) dominated by *Pinus roxburghii* trees, while the northernmost areas possess Himalayan moist temperate forest (1500–3000 m) dominated by *Pinus wallichiana* and *Pinus roxburghii*. The wetlands throughout this range exist in the form of freshwater streams [29]. *A*. *hazarensis* is endemic to Pakistan while *N*. *vicina* is known from Pakistan and India. *A*. *hazarensis* and *N*. *vicina* are known from streams and pools in forested mountainous areas as high as 1500 m [68] and 3000 m, respectively [64]. During our study, we found that *A*. *hazarensis* could occur at a higher elevation (>1500) than the previously reported range.

## Conservation implications and suggestions

Urban developments have been shown to have negative impacts on amphibians [69,70]. Several types of culverts, tunnels and corridors have been developed and their effectiveness assessed. Many amphibian species in North America and Europe have used these structures, resulting in reduced mortality from vehicular collisions [71]. Since anurans in Pakistan enjoy no legal protection, no such consideration is given during urban planning. With recent urban expansion in Rawalpindi District and the Islamabad Capital Territory [44,66], there has been a noticeable reduction in forests, open spaces and watersheds [72]. As shown by our results, some of the anuran study species are tolerant to habitat degradation while others are not. The creation of human modified habitats may further facilitate the spread of native species such as *Euphlyctis* spp., while also accommodating invasive species such as *D*. *bengalensis* and *D*. *stomaticus* which may pose the threat of resource competition against native species. We recommend studying the effectiveness of such existing amphibian tunnels and corridors or designing new ones tailored to the needs of our species. These could then be incorporated in future urban development programs.

Conservationists put more emphasis on conserving threatened species. Studies, however, have shown that even common species are subjected to population decline and local extinction especially if these species are associated with a particular type of habitat or set of environmental conditions [73,74]. A 29% decrease in the population of the Moor Frog (*Rana arvalis*), which is found in heathlands and moorlands in parts of Europe, was reported between 1950–2006 [73]. This decline was attributed to cultivation of heathlands and moorlands, lowering of ground water levels and intensification of agricultural practices. In a forest landscape study, Brown Frogs (*Rana arvalis*, *R*. *temporaria*) bred more in various wetland habitats (e. g., naturally flooded areas, beaver ponds, mitigation pools with shallow littoral zones, cleaned ditches) than in ditches overgrown with forest vegetation [74].

Climate change during the past two decades has affected several species of plants and animals in Pakistan [75]. The tadpoles of *A*. *hazarensis* and *N*. *vicina* responded (under laboratory conditions) to higher temperature (*>*26°C) through faster metamorphosis, reduction in the body size, more frequent developmental complications or deformities such as edema and tail kinks, lower fitness and higher mortality [76]. Being associated with a particular set of environmental conditions in the north and northeast of the study area, it is feared that these two species endemic frogs, which are currently evaluated as least concern in the IUCN Red List of threatened species [77], may experience local extinction in the future. Land use simulators can be used to project changes over space and time in environmental variables used both in Maxent models (e.g., climate) and in models based on surveys whose locations were informed by Maxent model predictions. Thus, Maxent models can be used to project changes in distribution of species over time under different climate scenarios, and raster layers based on these distributions can be used to identify potential refugia for anurans either under current or future climate conditions [78]. These raster layers may also be used as inputs in raster overlay-based conservation planning tools (e.g., Marxan, Zonation) to prioritise locations for protection or management of threatened species [79].

## Supporting information

S1 File GBIF occurrences. (CSV)

S2 File Derived GBIF dataset. (TXT)

S3 File Maxent Output for *Allopaa hazarensis*. (PDF)

S4 File Maxent Output for *Duttaphrynus bengalensis*. (PDF)

S5 File Maxent Output for *Duttaphrynus stomaticus*. (PDF)

S6 File Maxent Output for *Euphlyctis* spp. (PDF)

S7 File Maxent Output for *Hoplobatrachus tigerinus*. (PDF)

S8 File Maxent Output for *Microhyla nilphamariensis*. (PDF)

S9 File Maxent Output for *Minervarya* spp. (PDF)

S10 File Maxent Output for *Nanorana vicina*. (PDF)

S11 File Maxent Output for *Sphaerotheca pashchima*. (PDF)
